# Editorial: Inter-healthcare Professions Collaboration: Educational and Practical Aspects and New Developments

**DOI:** 10.3389/fphar.2016.00306

**Published:** 2016-09-12

**Authors:** Nancy F. Fjortoft, Susan Cornell, Mary A. Kliethermes, Lon J. Van Winkle

**Affiliations:** ^1^Department of Pharmacy Practice, Chicago College of Pharmacy, Midwestern UniversityDowners Grove, IL, USA; ^2^Department of Biochemistry, Midwestern UniversityDowners Grove, IL, USA

**Keywords:** inter-professional collaboration, healthcare professions students, patient-centered medical homes, team-based care, patient-centered care

NF is a 62 year old female patient in good health. She is seeing her family physician for routine care, including managing her high blood pressure, annual check-ups, immunizations, and standard screenings. She also sees a podiatrist regarding a heel spur on her right foot, a GI specialist for GERD, an ophthalmologist for follow-up care after cataract surgery, a physical therapist for left knee pain, a dentist for a new crown, and her pharmacist manages all of her medications. She has a preferred provider organization (PPO) insurance plan so she can select her own care givers without going through her primary care physician. As a result, her “team” does not share any network, nor do they share an electronic medical record. Is her investment in health care seeing optimal results? Who is managing her care? The likely answer to this question is no one and everyone.

The United States spends more per capita on health care than any other nation, yet ranks below other comparable countries on key criteria such as access, efficiency, and most importantly, health outcomes (Davis et al., 2014). For a primary care physician to take care of all needs, chronic and preventative, for an average panel of 2500 patients, they would need to work 21 h a day (Altschuler et al., 2012). Clearly this practice is not sustainable, nor is it seeing optimal results.

Interprofessional collaboration, in particular, in patient-centered medical homes, is designed to deliver high-quality, team-based, patient-centered primary care. Evidence indicates that this approach increases health outcomes, in particular, those related to chronic diseases, and reduces emergency room visits, length of stay, specialty provider visits, all at tremendous cost savings (Nielsen et al., 2016).

How do we as educators prepare our students for this new world of team-based patient-centered care? Interprofessional education is described as occurring when two or more professions learn about, from and with each other to enable effective collaboration and improve health outcomes (Health Professions Networks Nursing and Midwifery Human Resources for Health, 2010). The interprofessional education collaborative identified four domains of core competencies for interprofessional collaborative practice: values/ethics for interprofessional practice; roles and responsibilities; interprofessional communication; teams and teamwork (Interprofessional Education Expert Panel, 2011).

A number of efforts to foster these core competencies are presented in this topic. For example, Tamayo et al. point out that, if efforts to improve empathy and attitudes toward interprofessional collaboration are included early in the curricula of healthcare professional students, they would likely foster work to reinforce the values for interprofessional practice later on. Van Winkle adds, that interdisciplinary teams of healthcare professional students reflecting together on their attitudes toward each other, helps to mitigate their biases toward one another. Although work needs to be done to help small numbers of healthcare professional students whose negative biases are increased by the experience (Van Winkle). In these ways, the roles and responsibilities of each healthcare professional are also clarified. Similarly, Stringer et al. remind us that we continue to need to learn more about the roles and responsibilities of other professionals. Clearly the work in teams used in these studies (e.g., Van Winkle; Van Winkle) fosters the communication needed to acquire all of the competencies listed above. Nevertheless, Wang and Zorek emphasize that a concerted effort of experiential learning followed by reflection followed by another round of learning should be implemented best to foster interprofessional education through deliberate practice theory. Thus, it is clear there remains much to be done. Interprofessional education is still in its infancy, but for true interprofessional collaboration in practice to occur, students must learn the four core domains in their academic programs.

Let's go back to NF our 62 year old patient and imagine how her care would be different if she was in a team-based patient-centered medical home.

- Her care is centered on her. She is treated as a person with a life not as a set of medical conditions. Her team communicates with each other and shares all information regarding her health. They are incentivized to make sure her blood pressure is controlled, that she is getting the services needed, and there is no duplication of services. Her team ensures that she understands her conditions, her medications and her role in her path to better health.
- NF is now receiving comprehensive care. Nothing gets missed because everyone is working together. She is able to develop long term relationships with her providers.
- NF is now receiving coordinated care. Her information is shared and communicated. Her team is coordinated, making all her clinic visits smooth and seamless. The team works with everyone connected to her health in her community and connects her with resources she may need.
- NF now has better access to care. When she needs someone or has a question she can get them answered in a timely manner.
- NF is experiencing a systems based approach to quality and safety. Her team is rewarded for helping NF reach her health goals, and NF saves money and achieves better health.

As educators, we have the privilege and the responsibility to prepare our students for practice today and tomorrow. We need to keep patient NF in mind and focus on teaching the four core competencies of values/ethics, roles and responsibilities, interprofessional communication, and teams and teamwork.

## Author contributions

Dr. NF was the lead author, with substantial contributions from Drs. MK and LV, and some contributions from Dr. SC.

### Conflict of interest statement

The authors declare that the research was conducted in the absence of any commercial or financial relationships that could be construed as a potential conflict of interest.
